# The Preparation and Evaluation of Carvacrol-Added Hyaluronic Acid for Early Osteoarthritis Treatment

**DOI:** 10.3390/antiox14101265

**Published:** 2025-10-21

**Authors:** Yu-Ping Chen, Jhih-Ni Lin, Chia-Tien Chang, Yu-Ying Lin, Che-Yung Kuan, Yu-Chun Chen, Feng-Huei Lin

**Affiliations:** 1 Doctoral Program in Tissue Engineering and Regenerative Medicine, National Chung Hsing University, Taichung 40227, Taiwan; asder89106@gmail.com (Y.-P.C.); aaiaaiaai0813@gmail.com (C.-T.C.); butter97132195@gmail.com (Y.-Y.L.); madkuan@gmail.com (C.-Y.K.); 2 Institute of Biomedical Engineering and Nanomedicine, National Health Research Institutes, Miaoli 35053, Taiwan; febe200630@gmail.com; 3 Department of Chemical Engineering, College of Engineering and Science, National United University, Miaoli 36063, Taiwan; 4 Department of Biomedical Engineering, College of Medicine and College of Engineering, National Taiwan University, Taipei 10617, Taiwan

**Keywords:** osteoarthritis, hyaluronic acid, carvacrol, interleukin-1β, anti-inflammation, antioxidant

## Abstract

Osteoarthritis (OA) is a prevalent degenerative joint disease characterized by cartilage degradation, synovial inflammation, and subchondral bone remodeling, leading to chronic pain and reduced mobility. In early-stage OA, sustained oxidative stress and inflammation drive chondrocyte dysfunction and extracellular matrix (ECM) loss. Hyaluronic acid (HA), a key component of synovial fluid responsible for lubrication and viscoelasticity, is prone to enzymatic and oxidative degradation under inflammatory conditions, limiting its therapeutic effect. To address this, we developed an HA-based system incorporating the natural antioxidant and anti-inflammatory molecule carvacrol. The potential of this formulation was assessed in interleukin-1b-stimulated chondrocytes, which mimic the inflammatory environment of OA. The carvacrol-added HA combination upregulated antioxidant enzyme expression, attenuated pro-inflammatory signaling, and promoted ECM preservation by up regulating cartilage-specific markers and glycosaminoglycan production. In vivo efficacy was further evaluated in a rat model of monosodium iodoacetate-induced OA. HA-Carvacrol treatment alleviated pain-related behaviors and preserved cartilage structure, as confirmed by behavioral assessments and histological analyses. This dual-function formulation integrates the lubricating benefits of HA with the bioactivity of carvacrol, providing preclinical proof-of-concept evidence for its potential in early-stage OA.

## 1. Introduction

Osteoarthritis (OA) is a chronic joint disease that affects approximately 500 million individuals worldwide [1]. Among the various joints, the knee is not only the most commonly affected site [2], but is also particularly susceptible to OA development due to its complex anatomy and weight-bearing function [3]. The development and progression of OA are influenced by multiple risk factors, including physical activity, genetic predisposition, sex, obesity, and age [4]. These factors contribute to inflammation, oxidative stress, and structural changes in the joint tissues, all of which play critical roles in OA pathogenesis [5].

Inflammation, which is often initiated by joint trauma or overuse, drives cartilage degradation and joint deterioration [6]. Among pro-inflammatory mediators, interleukin-1β (IL-1β) is particularly prominent, with elevated levels observed in the synovial fluid, synovium, cartilage, and subchondral bone of patients with OA [7]. IL-1β promotes the release of interleukin-6 (IL-6) and tumor necrosis factor-α (TNF-α), and induces matrix metalloproteinases (MMPs), resulting in breakdown of the extracellular matrix (ECM) and the suppression of proteoglycan and collagen type II (Collagen II) synthesis [5,8]. In addition, IL-1b stimulation directly triggers excessive production of reactive oxygen species (ROS) through mitochondrial dysfunction [9,10,11], and both IL-1b and ROS have been implicated in the fragmentation of hyaluronic acid (HA) into low-molecular-weight forms, thereby enhancing pro-inflammatory pathways [12,13].

Oxidative stress is another critical contributor to OA pathophysiology. Elevated levels of ROS, including hydrogen peroxide, superoxide anions, hydroxyl radicals, and nitric oxide, have been detected in OA-affected joints [14]. This oxidative imbalance disrupts tissue homeostasis, decreases matrix synthesis, and accelerates cartilage degeneration [10,15]. HA, a major component of synovial fluid and the cartilage ECM, is essential for joint lubrication, viscoelasticity, and shock absorption [16]. Low-molecular-weight HA lacks the viscoelastic and protective functions of native HA and has been associated with impaired joint homeostasis and OA progression [17]. As OA progresses, pathological changes such as cartilage erosion, joint space narrowing, subchondral bone sclerosis, and osteophyte formation become evident, leading to joint dysfunction and chronic pain [18,19,20].

Current intra-articular therapies for OA include corticosteroids, platelet-rich plasma (PRP), and HA injections [21]. Corticosteroids provide temporary symptom relief but may accelerate cartilage degeneration with repeated administration [22,23]. Autologous blood-derived PRP helps reduce inflammation and promote tissue regeneration, although its long-term efficacy remains uncertain [24,25]. HA injections provide pain relief and functional improvement in early-stage OA by enhancing lubrication and joint mechanics, particularly when using high-molecular-weight formulations that provide better viscoelasticity and joint retention [26,27,28]. However, the long-term clinical benefits of HA remain debated, as it typically offers only temporary relief of OA-related symptoms and has limited effects on disease progression. Moreover, due to the lack of intrinsic anti-inflammatory, antioxidant, and tissue-regenerative bioactivity, HA alone is insufficient to modulate the underlying pathophysiological mechanisms of OA [29,30,31].

Synergistic intra-articular strategies that combine joint lubrication with the therapeutic modulation of joint pathology represent a promising approach for early-stage OA treatment, particularly for relieving pain, synovial inflammation, and cartilage degeneration [32]. Among these, supplementing HA with functional natural compounds, particularly those with anti-inflammatory and antioxidant properties, has gained attention as a means of enhancing therapeutic efficacy [31,33]. Phytochemicals, especially those derived from medicinal herbs, have been widely explored due to their multifaceted mechanisms of action, favorable safety profiles, and cost-effectiveness [34,35]. Among these, phenolic compounds commonly found in medicinal plants exhibit antioxidant activity comparable to that of synthetic antioxidants [36,37]. In addition to scavenging ROS, phenolics modulate key inflammatory and metabolic signaling pathways implicated in OA pathogenesis [5,38]. This dual functionality not only contributes to symptomatic relief but also offers potential for disease modification. Notably, several phenolic compounds have demonstrated protective effects on cartilage by attenuating oxidative stress and suppressing pro-inflammatory mediators in OA-related in vitro and in vivo models [39,40].

Representative examples include curcumin [41], resveratrol [42], and quercetin [43], all of which have been extensively studied for their anti-inflammatory and antioxidant activities in joint- and cartilage-related conditions. Carvacrol, a monoterpenoid phenol found in oregano and thyme, has emerged as a promising candidate. Preclinical studies have demonstrated the anti-inflammatory and antioxidant effects of carvacrol in various chronic inflammatory and autoimmune disease models [44,45,46,47,48]. In a preclinical study, carvacrol was shown to suppress inflammatory responses by downregulating IL-1b signaling and enhancing antioxidant defenses by promoting endogenous antioxidant enzymes [47]. In a preclinical study of rheumatoid arthritis induced by lipopolysaccharide (LPS), carvacrol reduced the expression of matrix-degrading enzymes and pro-inflammatory cytokines by inhibiting the TLR4/MyD88/NF-kB and MAPK signaling pathways [48]. In addition to preclinical studies, clinical investigations have indicated that carvacrol is safe, well-tolerated, and capable of modulating oxidant/antioxidant balance in humans [49,50]. Nevertheless, the current body of evidence is still limited; preclinical studies are restricted to specific models, and clinical research lacks randomized controlled trials and meta-analyses, indicating that additional preclinical studies are required to strengthen the evidence.

The mechanisms of carvacrol are closely associated with the pathological features of early-stage OA, which include elevated levels of inflammatory cytokines and oxidative stress [5]. Although previous studies have reported anti-inflammatory and antioxidant properties of carvacrol in various models, direct evidence in the context of OA pathogenesis remains limited. In this regard, incorporating carvacrol into HA has not been extensively investigated, particularly for intra-articular applications. In the context of conjugation with HA, phenolic hydroxyl groups are often involved as reactive functional groups [51]. The presence of the phenolic –OH group in carvacrol is the major reason for its radical scavenging activity [52]; therefore, we avoided cross-linking strategies that could consume this group. In this study, we prepared an HA–carvacrol formulation using a simple approach, combining the lubricating properties of HA with the anti-inflammatory and antioxidant effects of carvacrol, and performed a proof-of-concept evaluation in early-stage OA by assessing its effects on inflammation, oxidative stress, and cartilage protection.

## 2. Materials and Methods

### 2.1. Preparation of HA-Carvacrol Solution

Hyaluronic acid (HA; MW = 1.7 × 10^6^ Da; Kewpie, Tokyo, Japan) was dissolved in minimum essential medium alpha (α-MEM; Gibco, Waltham, MA, USA) under continuous stirring until fully solubilized. Carvacrol (Sigma-Aldrich, St. Louis, MO, USA) was subsequently added to the HA solution at a final concentration of 10 μg/mL. Although carvacrol has limited aqueous solubility, the mixture was macroscopically uniform after mixing at this low concentration [53,54]. The solution was immediately filtered through a 0.22 mm membrane into sterile vials and freshly prepared before each experiment.

### 2.2. Free Radical Scavenging Assay

A 2,2-diphenyl-1-picrylhydrazyl (DPPH; Sigma-Aldrich, USA) assay was performed to evaluate the free radical scavenging activity of the samples. A 200 μM DPPH stock solution was prepared in 95% ethanol. Equal volumes of the DPPH solution and each test sample were mixed in a 96-well plate. Phosphate-buffered saline (PBS) was used as a blank control, and ascorbic acid served as a positive control. Absorbance was measured at 517 nm using a microplate reader (BioTek^®^, Xinbei, Taiwan). The scavenging activity was calculated based on the reduction in absorbance relative to that of the blank control.

### 2.3. Cell Culture and Treatment

To establish an in vitro inflammatory model, human chondrocytes C20A4 cells (Cat #SCC041, male; Merck, Darmstadt, Germany) were cultured in α-MEM supplemented with 10% fetal bovine serum (Gibco, USA) and 1% antibiotic–antimycotic (Gibco, USA). The cells were maintained at 37 °C in a humidified incubator with 5% CO_2_. After 24 h of incubation, inflammation was induced by adding IL-1β (10 ng/mL; Asia Bioscience, Taipei City, Taiwan). The following day, the cells were treated with HA or HA-Carvacrol.

### 2.4. Chondrocyte Viability Under Inflammatory Conditions

The WST assay was used to quantitatively assess cellular metabolic activity as an indicator of cell viability. To determine the optimal working concentration of carvacrol, cells were first treated with different concentrations to evaluate cell viability. IL-1b was then applied to mimic the inflammatory conditions characteristic of osteoarthritis. C20A4 cells were stimulated with IL-1β (10 ng/mL) for 24 h, followed by treatment with HA or HA-Carvacrol. Untreated cells served as the negative control, whereas cells exposed to 0.01% Triton X-100, a known cytotoxic agent, served as the positive control. On days 1 and 3 post-treatment, the WST reagent (Elabscience, Houston, TX, USA) was added to each well, and absorbance was measured at 450 nm using a microplate reader (BioTek^®^, Taiwan). Higher absorbance values indicate increased mitochondrial dehydrogenase activity, reflecting greater cell viability.

In parallel, a qualitative assessment of cell viability was conducted using live/dead staining to evaluate membrane integrity. On days 1 and 3 post-treatment, calcein-AM and ethidium homodimer-1 (EthD-1) (Invitrogen, Waltham, MA, USA) were applied according to the manufacturer’s protocol under the same stimulation and treatment conditions. Fluorescent images were captured using an inverted fluorescence microscope (Eclipse Ts2-FL, Nikon, Tokyo, Japan), with live cells fluorescing green and dead cells fluorescing red. Quantitative analysis of live/dead images was performed using ImageJ by calculating the percentage of viable cells (green) relative to the total cell number. Experiments were performed in six replicates (*n* = 6).

### 2.5. Chondrocyte Cytotoxicity Under Inflammatory Conditions

Lactate dehydrogenase (LDH) release into the culture supernatants was measured using the Cytotoxicity Detection Kit PLUS (Roche, Basel, Switzerland) to evaluate membrane damage and cytotoxicity. C20A4 cells were first stimulated with IL-1β (10 ng/mL) for 24 h, followed by treatment with HA or HA-Carvacrol. Negative controls were untreated cells, while positive controls were cells exposed to 0.01% Triton X-100. On days 1 and 3 post-treatment, LDH activity in the culture supernatants was quantified according to the manufacturer’s instructions. Absorbance was measured at 490 nm using a microplate reader. Experiments were performed in six replicates (*n* = 6).

### 2.6. Gene Expression Analysis of OA-Related Markers Under Inflammatory Conditions

Quantitative real-time PCR (qRT-PCR) was performed to evaluate the expression levels of genes related to inflammation, oxidative stress, and extracellular matrix regulation in IL-1β-stimulated chondrocytes treated with HA or HA-Carvacrol. C20A4 chondrocytes were stimulated with IL-1β (10 ng/mL) for 24 h, followed by treatment with the respective formulations. The cells were harvested on days 1 and 3 post-treatment for RNA extraction and gene expression analysis. Experiments were performed in six replicates (*n* = 6).

Total RNA was extracted using the GENEzol^TM^ TriRNA Pure Kit (Geneaid, New Taipei City, Taiwan), and RNA concentration and purity were determined using a micro-volume spectrophotometer (Nano-400A, Medclub Scientific, Taipei, Taiwan). Quantitative real-time PCR was performed using the KAPA SYBR^®^ FAST One-Step qRT-PCR Kit (KAPA Biosystems, Wilmington, MA, USA), which combines reverse transcription and amplification into a single reaction. Reactions were performed on a QuantStudio^TM^ 1 Real-Time PCR System (Thermo Fisher Scientific, Waltham, MA, USA). The thermal cycling protocol involved reverse transcription at 42 °C for 5 min, initial denaturation at 95 °C for 3 min, followed by 35 cycles of denaturation at 95 °C for 3 s and annealing/extension at 60 °C for 80 s.

Primers were designed to target genes related to inflammation (IL-1β, IL-6, TNF-α, and IL-1RA), oxidative stress (iNOS, GPX, SOD, and CAT), ECM regulation (Aggrecan, Collagen II, Versican, and Collagen I), and matrix degradation (MMP3 and MMP13). All primers were synthesized by PURIGO (Taipei, Taiwan), and the sequences are provided in Supplementary Table S1.

GAPDH was used as an internal control, and its Ct values remained stable across experimental groups. The Ct value of each target gene was normalized to GAPDH (ΔCt), and relative gene expression levels were evaluated using the –ΔΔCt method.

### 2.7. Quantification of Inflammation-Related Proteins Under Inflammatory Conditions

IL-6 levels in the culture supernatants were quantified using enzyme-linked immunosorbent assay (ELISA) to evaluate the inflammatory response. C20A4 chondrocytes were stimulated with IL-1β (10 ng/mL) for 24 h, followed by treatment with HA or HA-Carvacrol. On days 1 and 3 post-treatment, the supernatants were collected and analyzed using a commercial ELISA kit (Elabscience, USA) according to the manufacturer’s instructions. Absorbance was measured at 450 nm using a microplate reader (BioTek^®^, Taiwan), and IL-6 concentrations were calculated based on a standard curve. Experiments were performed in six replicates (*n* = 6).

### 2.8. Quantification of Cartilage Matrix Molecules Under Inflammatory Conditions

The glycosaminoglycan (GAG) content in the culture supernatants was quantified using the dimethylmethylene blue (DMMB) assay to assess extracellular matrix production. C20A4 chondrocytes were stimulated with IL-1β (10 ng/mL) for 24 h, followed by treatment with HA or HA-Carvacrol. On days 1 and 3 post-treatment, culture supernatants were collected and mixed with the DMMB dye solution. Absorbance was measured at 595 nm using a microplate reader (BioTek^®^, Taiwan). GAG concentrations were calculated from a standard curve prepared with chondroitin sulfate diluted in α-MEM (0–50 μg/mL). Experiments were performed in six replicates (*n* = 6).

### 2.9. Establishment of Monosodium Iodoacetate (MIA)-Induced OA Model in Rats

To evaluate the therapeutic efficacy and safety of HA and HA-Carvacrol formulations in an MIA-induced OA model, male Sprague-Dawley rats (6–8 weeks old; LASCO, Taipei, Taiwan) were used. OA was induced on day 0 via a single intra-articular injection of 0.3 mg MIA in 50 μL sterile saline into the right knee joint under isoflurane anesthesia. This procedure induces chondrocyte death and cartilage degeneration, replicating the pathological features of human OA. All animal procedures were approved by the Institutional Animal Care and Use Committee of the National Health Research Institutes (NHRI-IACUC-112118-S01).

Rats were randomly divided into five groups (n = 6 per group): Control (no injection), Sham (PBS only), MIA (0.3 mg/50 μL), HA (1%), and HA-Carvacrol (1% HA containing 10 μg/mL carvacrol). All solutions for intra-articular injection (PBS, MIA, HA, and HA-Carvacrol) were sterilized by passage through a 0.22 μm membrane filter and freshly prepared before use. Intra-articular injections were administered once per week on days 3, 10, and 17. No further treatment was provided to allow the therapeutic effects to manifest without interference from the acute pharmacological activity. On day 31, all animals underwent behavioral testing to assess pain-related responses. Subsequently, rats were euthanized for blood collection and joint harvesting. Hematological and serum biochemical analyses were performed, and knee joint tissues were processed for the histological evaluation of cartilage integrity and inflammation. Group allocation was randomized, and blinding was ensured by assigning drug administration and behavioral assessments to different personnel.

### 2.10. Body Weight Monitoring

In animal OA experiments, changes in the body weight are important monitoring indicators. Body weight was measured prior to the start of the experiment to establish a baseline and was continued throughout the study period. Monitoring body weight allowed the assessment of general health status and potential physiological changes during treatment in the OA model.

### 2.11. Evaluation of Hematological and Biochemical Parameters

Blood samples were collected from rats at week 4 post-treatment and were submitted to the NHRI Animal Center (Miaoli, Taiwan) for safety evaluation. For hematological analysis, blood was drawn into ethylenediaminetetraacetic acid-treated tubes and mixed gently. Parameters including red blood cells (RBC), hemoglobin (HGB), hematocrit (HCT), mean corpuscular volume (MCV), mean corpuscular hemoglobin (MCH), mean corpuscular hemoglobin concentration (MCHC), platelets (PLT), white blood cells (WBC), monocytes (MO), and lymphocytes (LY) were analyzed using a veterinary hematology analyzer (Mindray BC-5000 Vet, Shenzhen, China). For biochemical analysis, serum was obtained by centrifugation at 3000× *g* for 15 min., and markers of liver function (AST, ALT, and ALP) and kidney function (BUN and CRE) were measured using a dry chemistry analyzer (Fuji DRI-CHEM NX500i, Tokyo, Japan).

### 2.12. Assessment of Mechanical Allodynia

Mechanical allodynia in OA rats was assessed using an electronic von Frey apparatus (IITC Life Science, Carlsbad, CA, USA). Each animal was placed on a wire-mesh platform within a transparent enclosure for acclimation. A gradually increasing force was applied to the mid-plantar surface of the right hind paw, and the force (g) required to induce a paw withdrawal response was recorded as the paw withdrawal threshold (PWT). Each rat received three stimulations at 5 min intervals, and the average value was used for analysis.

### 2.13. Thermal Hyperalgesia Test Using Hot Plate Method

Thermal hyperalgesia was evaluated using the hot plate test. Rats were individually placed on a heated surface maintained at 53 °C (SA705, SansBio, Nanjing, China), and the latency to the first pain-related behavior, such as paw licking or jumping, was recorded as the paw withdrawal latency (PWL). A cutoff time of 30 s was used to prevent tissue damage. Each rat was tested three times with 10 min intervals between trials, and the average value was calculated.

### 2.14. Histological Examination of Articular Cartilage

Structural alterations in the joint tissues were assessed histologically. The knee joints were harvested using sterile instruments, trimmed from the surrounding soft tissues, and rinsed with PBS. The samples were fixed in 10% neutral buffered formalin, decalcified in EDTA, and embedded in paraffin using an automated embedding system. All tissue processing and staining procedures were conducted at the Pathology Core Laboratory of NHRI (Miaoli, Taiwan). Paraffin sections were deparaffinized in xylene, rehydrated using graded ethanol, and stained with hematoxylin and eosin (H&E) to visualize the tissue morphology. The slides were subsequently dehydrated, cleared, and mounted with a resin-based medium for microscopic examination.

### 2.15. Statistical Analysis

All experiments were conducted independently, and data are presented as mean ± standard deviation (SD). Data analysis was performed using ImageJ (version 1.54d) (NIH, Bethesda, MD, USA) and GraphPad Prism 8.0 (GraphPad Software, San Diego, CA, USA). Normality was examined using the Shapiro–Wilk test. Statistical significance was determined using one-way analysis of variance (ANOVA), with *p* < 0.05 considered statistically significant.

## 3. Results

### 3.1. Antioxidant Activity of HA-Carvacrol

**Figure 1:**
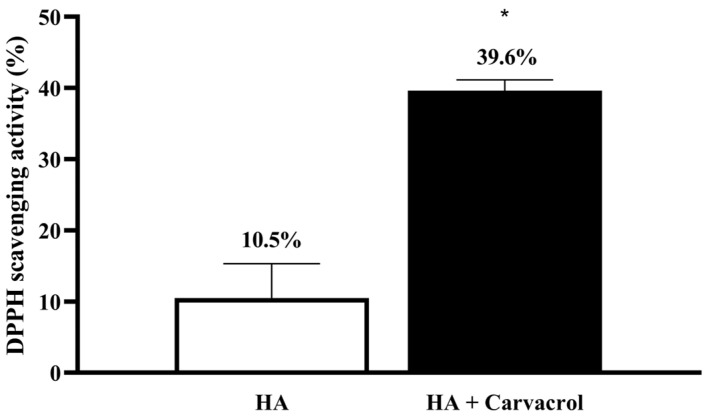
Carvacrol enhances the antioxidant capacity of HA. The HA-Carvacrol formulation exhibited significantly higher DPPH radical scavenging activity than HA alone, indicating improved antioxidant potential. Data are presented as mean ± SD (*n* = 6). * *p* < 0.05 compared with the HA group.

To determine whether the incorporation of carvacrol enhances the antioxidant properties of HA, a DPPH radical scavenging assay was performed. DPPH is a stable nitrogen-centered free radical that is widely used to evaluate the antioxidant capacity via electron transfer mechanisms [55]. As shown in Figure 1, HA alone exhibited a low DPPH scavenging rate, whereas the HA-Carvacrol formulation significantly increased the scavenging activity. Compared with the standard curve of ascorbic acid used as a positive control (Supplementary Figure S1), the scavenging activity of the HA–carvacrol formulation corresponded to approximately 6.3 mg/mL of ascorbic acid, providing a reference for interpreting its antioxidant capacity. These results demonstrated that carvacrol incorporation markedly upregulated the antioxidant activity of the HA formulation.

### 3.2. Cytocompatibility and Cytotoxicity Under Inflammatory Conditions

**Figure 2:**
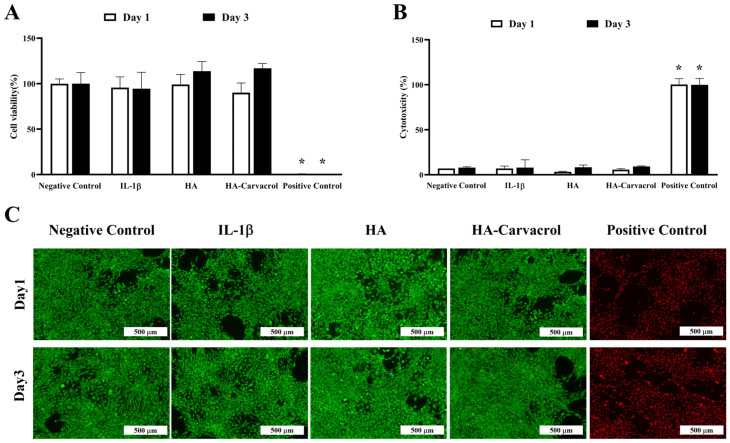
HA-Carvacrol preserves chondrocyte viability and membrane integrity under IL-1β stimulation. (**A**) WST assay showed that HA-Carvacrol maintained metabolic activity in IL-1β-stimulated C20A4 cells. (**B**) LDH release assay demonstrated reduced membrane damage following HA-Carvacrol treatment. (**C**) Live/dead staining revealed a higher proportion of viable cells (green, calcein-AM) and fewer dead cells (red, EthD-1) in the HA-Carvacrol group. Scale bar = 500 μm. Data are presented as mean ± SD (*n* = 6). * *p* < 0.05 compared with the negative control group on the same day.

To ensure the appropriate working dose, different concentrations of carvacrol were first evaluated, and the results indicated that 10 mg/mL provided the best cell viability (Supplementary Figure S2). Based on this finding, subsequent experiments were conducted under inflammatory stress by stimulating C20A4 cells with IL-1b (10 ng/mL, 24 h). IL-1b is widely used in osteoarthritis research to establish in vitro models that mimic the inflammatory environment by inducing chondrocyte catabolic activity and inflammation [56,57]. Cell viability, membrane integrity, and cytotoxicity were then assessed via WST assay, LDH release, and live/dead staining on days 1 and 3 post-treatment. Under IL-1β stimulation, HA-Carvacrol treatment preserved chondrocyte viability, as evidenced by sustained metabolic activity in the WST assay (Figure 2A) and reduced LDH release, indicating minimal membrane damage (Figure 2B). Live/dead staining (Figure 2C) consistently revealed a predominance of viable (green-fluorescent) cells, supporting preserved membrane integrity and low cytotoxicity. Quantitative analysis of live/dead images confirmed that the HA-Carvacrol group showed no significant difference from the control group, indicating good cytocompatibility (Supplementary Figure S3). These findings suggested that HA-Carvacrol maintained cytocompatibility and effectively protected chondrocytes from IL-1β-induced inflammatory damage.

### 3.3. Effects on Inflammatory Molecular Responses in IL-1β-Stimulated Chondrocytes

Chronic inflammation plays a pivotal role in the early progression of OA, and elevated levels of pro-inflammatory cytokines contribute to cartilage degradation. Among these, IL-1β, IL-6, and TNF-α are key cytokines that induce matrix-degrading enzymes and amplify the inflammatory response [6]. To assess the anti-inflammatory potential of HA-Carvacrol, we evaluated both the gene expression and protein secretion of inflammatory cytokines in IL-1β-stimulated C20A4 cells.

**Figure 3:**
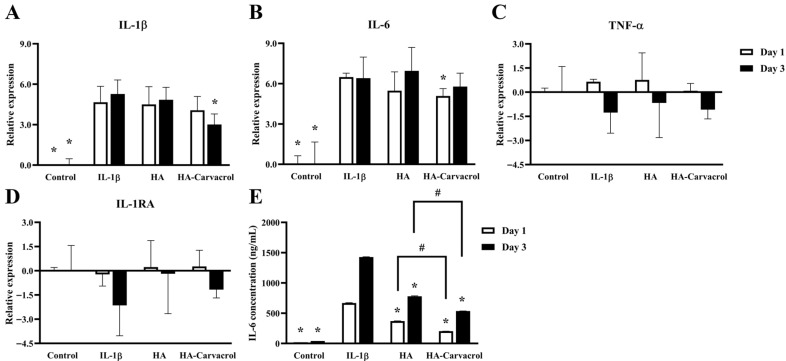
Effects of HA-Carvacrol on pro-inflammatory cytokines and IL-1RA expression in IL-1b-stimulated chondrocytes. (**A**–**C**) HA-Carvacrol treatment significantly reduced the mRNA expression of IL-1β, IL-6, and TNF-α. (**D**) IL-1RA expression, which was suppressed by IL-1β, was partially restored. (**E**) ELISA showed that IL-6 secretion was significantly decreased following HA-Carvacrol treatment. Data are presented as mean ± SD (*n* = 6). * *p* < 0.05 compared with the IL-1β group on the same day. # *p* < 0.05 compared with the HA group on the same day.

As shown in Figure 3A–C, IL-1β stimulation markedly upregulated the expression of IL-1β and IL-6, with a moderate increase observed in TNF-α. Treatment with HA or HA-Carvacrol suppressed IL-1β and IL-6 expression, with HA-Carvacrol exhibiting a more substantial reduction, particularly on day 3. The expression of the anti-inflammatory cytokine IL-1 receptor antagonist (IL-1RA), which was suppressed following IL-1β stimulation, was partially restored by treatment with HA or HA-Carvacrol. A similar upward trend was observed at both the time points (Figure 3D). In parallel, ELISA results showed that HA reduced IL-6 protein secretion by approximately 1.8-fold on both days 1 and day 3, while HA-Carvacrol achieved a more pronounced suppression of about 3.3-fold on day 1 and 2.7-fold on day 3. Taken together, these results suggest that HA-Carvacrol effectively suppressed IL-1β-induced inflammatory responses by downregulating pro-inflammatory cytokines and upregulating the anti-inflammatory cytokine IL-1RA.

### 3.4. Effects on Antioxidant Molecular Responses in IL-1β-Stimulated Chondrocytes

Oxidative stress plays a critical role in OA progression and is often exacerbated by inflammatory cytokines such as IL-1β. Elevated ROS levels impair chondrocyte viability and contribute to ECM degradation [10]. To investigate whether HA-Carvacrol could modulate oxidative stress, the expression levels of oxidative and antioxidant markers were examined in IL-1β-stimulated C20A4 cells.

**Figure 4:**
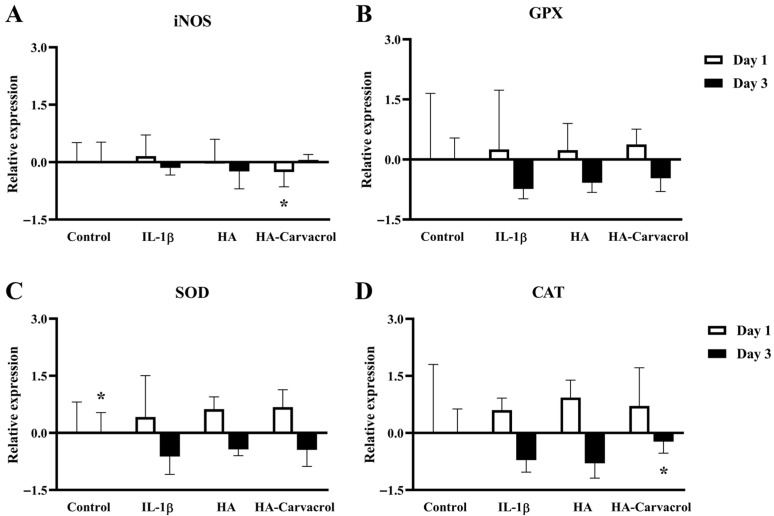
Effects of HA-Carvacrol on iNOS and antioxidant gene expression in IL-1β-stimulated chondrocytes. (**A**) HA-Carvacrol downregulated iNOS expression. (**B**–**D**) The expression levels of antioxidant enzymes SOD, CAT, and GPX were elevated, indicating upregulated endogenous antioxidant defense. Data are presented as mean ± SD (*n* = 6). * *p* < 0.05 compared with the IL-1β group on the same day.

As shown in Figure 4A, IL-1β stimulation upregulated iNOS expression. On day 1, both HA and HA-Carvacrol treatments reduced iNOS levels, with HA-Carvacrol exhibiting more pronounced suppression. The expression levels of antioxidant genes, including SOD, CAT, and GPX, were elevated following treatment (Figure 4B–D). This upregulation was observed on days 1 and day 3, and HA-Carvacrol consistently induced higher expression levels than HA alone. These findings suggest that HA-Carvacrol not only suppresses oxidative stress markers, such as iNOS, but also enhances endogenous antioxidant responses.

### 3.5. Effects on ECM-Related Molecular Responses in IL-1β-Stimulated Chondrocytes

Cartilage degradation in OA is primarily driven by an imbalance between matrix synthesis and degradation. Pro-inflammatory cytokines, such as IL-1β, suppress anabolic gene expression while upregulating catabolic enzymes, ultimately leading to ECM breakdown [58]. To evaluate the ECM-related responses of HA-Carvacrol, the expression of ECM-related genes and GAG content was examined in IL-1β–stimulated C20A4 cells.

**Figure 5:**
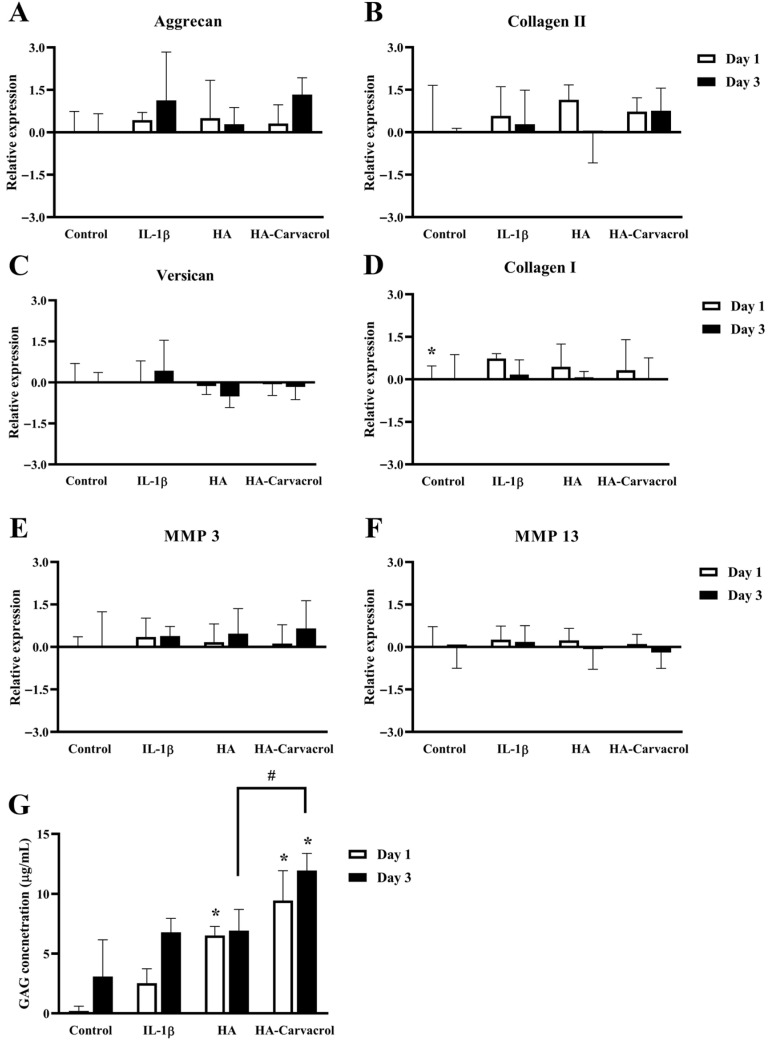
Effects of HA-Carvacrol on ECM synthesis and matrix degradation under inflammatory stress. (**A**,**B**) Expression of anabolic markers Aggrecan and Collagen II showed higher levels in the HA-Carvacrol group compared with the IL-1b group. (**C**,**D**) Expression of ECM remodeling-associated genes Versican and Collagen I showed lower levels in the treatment groups. (**E**,**F**) Catabolic enzymes MMP-3 and MMP-13 showed a decreasing trend following treatment. (**G**) GAG accumulation was significantly increased in the HA-Carvacrol group compared with the IL-1b group. Data are presented as mean ± SD (*n* = 6). * *p* < 0.05 compared with the IL-1β group on the same day; # *p* < 0.05 compared with the HA group on the same day.

As shown in Figure 5A,B, on day 3 the HA-Carvacrol group showed higher expression levels of Aggrecan and Collagen II compared with both the HA and IL-1b groups, but the differences were not statistically significant. These anabolic markers are essential for matrix synthesis, and their upward trend suggests a partial preservation of chondrocyte function. Expression of Versican and Collagen I was lower in both treatment groups compared with the IL-1b group (Figure 5C,D), markers that are commonly associated with matrix remodeling. MMP-3 and MMP-13 are catabolic enzymes that degrade proteoglycans and collagen II in cartilage. After treatment, their expression was lower in the treatment groups, but the differences were not statistically significant (Figure 5E,F).

To further support these findings, GAG content was measured in the culture supernatants. On both days 1 and 3, GAG levels increased in the treatment groups, with HA-Carvacrol showing a significant elevation compared with the IL-1b group (Figure 5G). These results suggest that HA-Carvacrol may help maintain cartilage matrix homeostasis under inflammatory conditions by increasing anabolic gene expression, reducing remodeling-related and catabolic-related markers and significantly elevating GAG accumulation.

### 3.6. In Vivo Biosafety Evaluation

An MIA-induced OA rat model was used to evaluate the in vivo biosafety of HA and HA-Carvacrol. The animals were divided into five groups: (1) Control, which received no injection; (2) Sham, which received an intra-articular injection of PBS; (3) MIA, which received 0.3 mg MIA to induce OA without treatment; (4) MIA + HA, treated with 1% HA; and (5) MIA + HA-Carvacrol, treated with 1% HA containing 10 mg/mL carvacrol.

**Figure 6:**
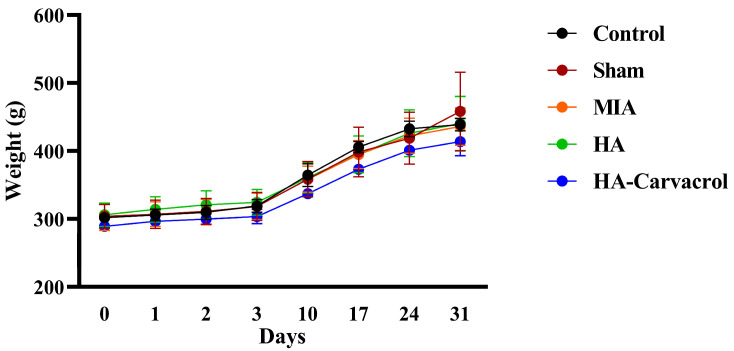
Body weight monitoring in monosodium iodoacetate (MIA)-induced osteoarthritic rats. Sham represents rats that received an intra-articular injection of PBS only. No significant differences in body weight were observed among groups throughout the experimental period, suggesting that HA or HA-Carvacrol treatment did not induce systemic toxicity or growth impairment. Data are presented as mean ± SD (*n* = 6).

Body weight, hematological parameters, and serum biochemical markers were monitored throughout the study to assess systemic biosafety. As shown in Figure 6, all groups exhibited comparable trends in body weight gain, suggesting that neither MIA induction nor intra-articular administration of HA or HA-Carvacrol adversely affected general health status. Hematological and biochemical analyses revealed no significant differences between the treatment and control groups. These results indicated that intra-articular administration of HA or HA-Carvacrol did not trigger systemic inflammation or cause liver and kidney dysfunction (Table 1). Overall, both HA and HA-Carvacrol were well tolerated in vivo, with no observable adverse effects on body weight, immune cell profiles, or liver and kidney functions in MIA-induced OA rats.

### 3.7. Behavioral Assessments and Histological Evaluation

Pain perception is a key indicator of OA severity and progression and is often evaluated through mechanical and thermal sensitivity tests in preclinical models. To assess the analgesic effects of HA-Carvacrol in vivo, MIA-induced OA rats were subjected to behavioral assessments, including PWT and PWL tests.

**Figure 7:**
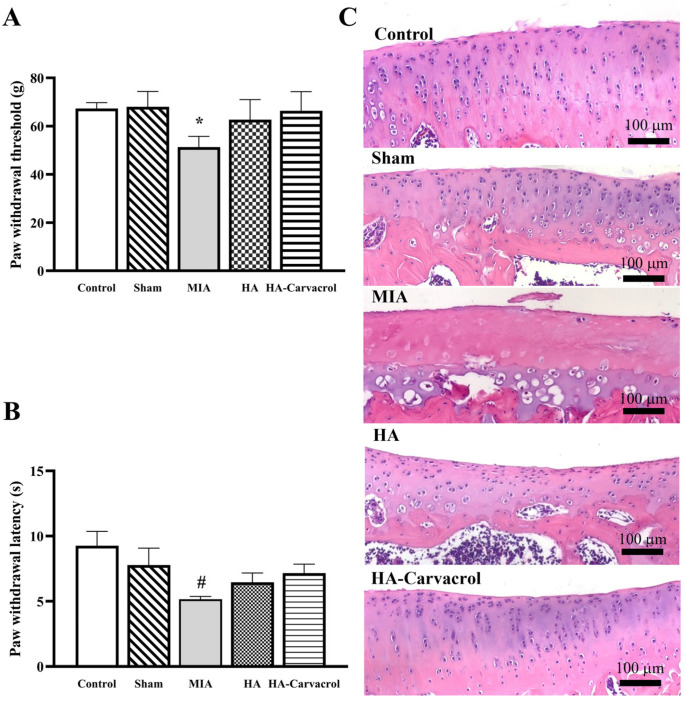
HA-Carvacrol alleviated pain behaviors and preserves cartilage structure in MIA-induced osteoarthritic rats. (**A**,**B**) HA-Carvacrol improved PWT and PWL, indicating reduced mechanical and thermal sensitivity. (**C**) H&E staining revealed better cartilage preservation in HA-Carvacrol-treated joints, with smoother surfaces and higher cellularity than the MIA group. Data are presented as mean ± SD (*n* = 6). * *p* < 0.05 compared with the MIA group, excluding the HA group. # *p* < 0.05 all groups compared with the MIA group.

As shown in Figure 7A,B, MIA injection significantly reduced both PWT and PWL, indicating the development of mechanical allodynia and thermal hyperalgesia. Treatment with HA or HA-Carvacrol improved both measures, with the HA-Carvacrol group showing a greater recovery in pain sensitivity. By the end of the experiment, behavioral responses in the HA-Carvacrol group were comparable to those in the control group, suggesting effective analgesic action under inflammatory conditions.

Histological analysis of knee joints using H&E staining (Figure 7C) further supported these findings. The MIA group exhibited pronounced cartilage damage, including surface irregularities and decreased chondrocyte density. In contrast, both HA and HA-Carvacrol treatments preserved the cartilage structure, with the HA-Carvacrol group showing more organized surface morphology and improved cellularity.

These results indicate that HA-Carvacrol not only mitigated OA-related pain but also preserved cartilage integrity, supporting its potential as a joint-protective therapeutic agent.

## 4. Discussion

Osteoarthritis is a progressive degenerative joint disorder characterized by cartilage breakdown, persistent inflammation, oxidative stress, and joint pain [59,60]. In this study, a carvacrol-containing HA formulation (HA-Carvacrol) was developed by direct mixing and evaluated through in vitro assays on chondrocytes and in vivo testing in an MIA-induced OA rat model.

Oxidative stress is a critical pathological factor in OA that accelerates cartilage degradation by promoting chondrocyte apoptosis and matrix breakdown through the excessive accumulation of reactive oxygen species (ROS) [11,15]. The DPPH assay confirmed the radical-scavenging ability of HA-Carvacrol, which was attributed to the phenolic hydroxyl group of Carvacrol, which donates hydrogen atoms to stabilize free radicals through resonance [52], supporting its direct antioxidant activity (Figure 1).

Pro-inflammatory cytokines such as IL-1β induce pro-oxidant enzymes, such as iNOS, leading to the excessive production of ROS and tissue damage [61,62]. HA-Carvacrol attenuated IL-1β-induced iNOS expression (Figure 4A) and upregulated antioxidant enzymes including SOD, CAT, and GPX (Figure 4B–D), crucial for ROS scavenging and intracellular redox balance [5,10,63]. By downregulating iNOS expression, HA-Carvacrol helps limit oxidative stress and protect cartilage integrity. These findings indicate that HA-Carvacrol scavenged extracellular radicals and may also contribute to the upregulation of endogenous antioxidant defenses in chondrocytes under inflammatory conditions.

In terms of cytocompatibility, HA-Carvacrol preserved chondrocyte viability under inflammatory stress (Figure 2A), as evidenced by the low LDH release and intact membrane integrity (Figure 2B,C). These results indicate the cytocompatibility of HA-Carvacrol and provide a basis for further research.

Pro-inflammatory cytokines such as IL-1β, IL-6, and TNF-α are critical mediators in the early stages of OA, contributing to cartilage degradation by stimulating matrix-degrading enzymes, amplifying inflammatory cascades, and disrupting chondrocyte homeostasis [5,7]. In this study, HA-Carvacrol treatment effectively attenuated IL-1β-induced inflammation in C20A4 chondrocytes by downregulating the expression of IL-1β, IL-6, and TNF-α (Figure 3A–C). Because IL-6 is a key cytokine involved in IL-1β-induced cartilage inflammation [64,65], the marked suppression of IL-6 secretion (Figure 3E) further supports the anti-inflammatory efficacy of HA-Carvacrol at the protein level. Additionally, HA-Carvacrol upregulated the expression of the anti-inflammatory cytokine IL-1RA, an endogenous inhibitor of IL-1 signaling (Figure 3D). As IL-1RA is critical for modulating inflammatory responses in joint tissues and its suppression is commonly associated with chronic inflammation in osteoarthritic cartilage [7], this partial restoration may reflect the therapeutic potential of HA-Carvacrol in re-establishing cytokine balance. Similar anti-inflammatory effects of carvacrol have been reported in various disease models [44,48]. Taken together, these findings suggest that HA-Carvacrol exerts anti-inflammatory effects in chondrocytes under inflammatory stimulation.

Cartilage homeostasis relies on the delicate balance between anabolic and catabolic activities. Under inflammatory stimulation, such as IL-1β exposure, this balance is disrupted by suppressed matrix synthesis and upregulated matrix-degrading enzymes, contributing to progressive cartilage breakdown [20,58,66]. In this study, HA-Carvacrol upregulated key anabolic genes, including Aggrecan and Collagen II (Figure 5A,B), and upregulated GAG content (Figure 5G), suggesting a potential role in promoting matrix synthesis and protecting against proteoglycan loss. As major ECM components, GAGs support cartilage elasticity, hydration, and compressive strength [67,68] and their elevation may indicate protection against inflammation-induced proteoglycan loss [66]. MMPs, particularly MMP-3 and MMP-13, are the key enzymes that degrade aggrecan and collagen II [69], and their upregulation is a central feature of OA progression closely linked to pro-inflammatory cytokine activity [70,71]. In the present study, HA-Carvacrol downregulated IL-1β-induced MMP-3 and MMP-13 expression (Figure 5E,F), which may imply reduced catabolic activity. Furthermore, HA-Carvacrol downregulated Versican and Collagen I (Figure 5C,D), two markers associated with aberrant cartilage remodeling [72,73]. Taken together, these results suggest that HA-Carvacrol may support matrix preservation and cartilage repair under inflammatory conditions.

In vitro, HA-Carvacrol showed antioxidant, anti-inflammatory, and chondroprotective effects, including maintained cell viability, extracellular matrix production, and the upregulation of anabolic and antioxidant markers together with the downregulation of catabolic and inflammatory markers. To further evaluate these effects in vivo, we used an MIA-induced OA rat model. In vivo assessments confirmed the biosafety and therapeutic potential of the HA-Carvacrol formulation. No adverse effects on body weight, hematological parameters, or serum biochemical markers were observed, indicating good systemic tolerance (Figure 6 and Table 1). Behavioral assessments confirmed that MIA-induced OA rats developed pronounced mechanical allodynia and thermal hyperalgesia, consistent with previous reports indicating dose-dependent increases in pain sensitivity following MIA injections [74]. Treatment with HA or HA-Carvacrol alleviated these pain responses, with the HA-Carvacrol group exhibiting greater improvements in PWT and PWL (Figure 7A,B). These effects are likely related to the compound’s anti-inflammatory activity, as inflammation is a key contributor to nociceptive sensitization and pain perception in OA [75,76]. Histological evaluation of knee joints further supported these findings. Compared to the severe cartilage degeneration observed in the MIA group, including surface erosion, disorganized chondrocyte arrangement, and matrix loss, treatment with HA-Carvacrol better preserved cartilage integrity. The HA-Carvacrol group exhibited smoother cartilage surfaces, higher chondrocyte densities, and clearer columnar architectures (Figure 7C), suggesting improved structural maintenance under inflammatory stress.

In summary, HA-Carvacrol alleviated pain and helped maintain cartilage structure in vivo, supporting its therapeutic potential for OA. As a preliminary proof-of-concept study, certain limitations remain, including the use of a single chondrocyte line in vitro, stimulation with only IL-1b, the absence of protein-level validation, and the lack of long-term efficacy and pharmacokinetic evaluations. Future studies should address these aspects through broader models and extended assessments, which will be essential to strengthen the preclinical evidence for HA–Carvacrol. Collectively, our findings provide preliminary evidence that HA–Carvacrol exerts antioxidant, anti-inflammatory, and ECM-protective effects in this proof-of-concept study. Future studies are warranted to validate these results and to strengthen the preclinical basis of HA–Carvacrol for early OA.

## 5. Conclusions

This study suggests that HA–Carvacrol, a hyaluronic acid formulation with the natural antioxidant carvacrol, shows potential in preclinical models of early-stage osteoarthritis. In both in vitro and in vivo models, HA-Carvacrol consistently attenuated oxidative stress and inflammation, helped preserve ECM integrity, and alleviated pain-related behaviors. These findings provide an initial preclinical basis for further investigation of HA–Carvacrol in early OA.

## Figures and Tables

**Table 1:** Biochemical and hematological parameters for systemic biosafety evaluation. No significant differences were observed among experimental groups in hematological indices or serum biochemical markers, including liver and kidney function parameters. Data are presented as mean ± SD (*n* = 6).

Item	Unit	Control	Sham	MIA	HA	HA-Carvacrol
RBC	10^6^/mL	7.93 ± 0.18	7.81 ± 0.73	7.77 ± 0.33	7.82 ± 0.1	7.89 ± 0.1
HGB	g/dL	15.6 ± 0.36	15.83 ± 0.83	15.8 ± 0.81	16.03 ± 0.25	16.06 ± 0.23
HCT	%	42.77 ± 0.55	43.17 ± 1.83	42.25 ± 1.73	43.37 ± 0.85	43.94 ± 0.5
MCV	fL	53.9 ± 1.85	55.47 ± 3.07	54.43 ± 0.31	55.47 ± 0.68	55.42 ± 1.12
MCH	pg	19.67 ± 0.84	20.37 ± 0.96	20.33 ± 0.26	20.5 ± 0.2	20.28 ± 0.34
MCHC	g/dL	36.47 ± 0.83	36.7 ± 0.4	37.38 ± 0.41	36.93 ± 0.15	36.66 ± 0.3
PLT	10^3^/mL	1023.67 ± 96.34	983.33 ± 424.51	1092.75 ± 114.21	1044.67 ± 223.31	1035 ± 142.95
WBC	10^3^/mL	11.05 ± 0.98	14.48 ± 2.4	13.44 ± 3.15	13.46 ± 4.08	11.17 ± 1.63
MO	10^3^/mL	0.69 ± 0.3	0.82 ± 0.23	0.72 ± 0.27	0.89 ± 0.41	0.57 ± 0.06
LY	10^3^/mL	8.86 ± 0.08	12.3 ± 2.6	11.3 ± 2.58	10.9 ± 3.65	9.1 ± 1.06
AST	U/L	106 ± 18.52	96.33 ± 23.97	93.75 ± 12.18	71.67 ± 10.79	76 ± 22.08
ALT	U/L	40 ± 7	40.33 ± 1.15	39.5 ± 8.35	35.67 ± 8.96	33.8 ± 3.9
ALP	U/L	442.33 ± 53.26	451.67 ± 51.03	422.5 ± 84.23	431 ± 74.36	546.4 ± 192.99
BUN	mg/dL	18.87 ± 2.18	18.7 ± 1.32	18 ± 2.82	17.17 ± 2.56	18.7 ± 1.35
CRE	mg/dL	0.29 ± 0.02	0.27 ± 0.03	0.27 ± 0.03	0.24 ± 0.04	0.25 ± 0.03

## Data Availability

The data presented in this study are available on request from the corresponding author.

## Supplementary Materials

The following supporting information can be downloaded at: https://www.mdpi.com/article/10.3390/antiox14101265/s1. Table S1. Primers for RT-PCR performance. Figure S1. Ascorbic acid was used as a positive control in the DPPH assay. The scavenging activity increased in a dose-dependent manner across the tested concentration range, confirming the validity of the assay. This standard curve was used as a reference to interpret the antioxidant capacity of the HA-carvacrol formulation. Data are presented as mean ± SD (*n* = 6). Figure S2. Cell viability of carvacrol at different concentrations in C20A4 cells evaluated by WST assay. The results demonstrated that 10 μg/mL provided the best cell viability and was therefore selected as the working concentration for subsequent experiments. Data are presented as mean ± SD (*n* = 6). * *p* < 0.05 compared with the negative control group on the same day. Figure S3. Quantitative analysis of live/dead staining in IL-1β-stimulated C20A4 chondrocytes. Live/dead assay indicated that HA-Carvacrol treatment maintained good cell viability under IL-1β stimulation. Data are presented as mean ± SD (*n* = 6). * *p* < 0.05 compared with the negative control group on the same day.

## Abbreviations

The following abbreviations are used in this manuscript:

OA	Osteoarthritis
MIA	Monosodium iodoacetate
ECM	Extracellular matrix
ROS	Reactive oxygen species
IL-1β	Interleukin-1β
IL-6	Interleukin-6
TNF-α	Tumor necrosis factor-α
IL-1RA	Interleukin-1 receptor antagonist
MMP	Matrix metalloproteinases
Collagen II	Collagen type II
Collagen I	Collagen type I
iNOS	Inducible nitric oxide synthase
SOD	Superoxide dismutase
CAT	Catalase
GPX	Glutathione peroxidase
RBC	Red blood cell
HGB	Hemoglobin
HCT	Hematocrit
MCV	Mean corpuscular volume
MCH	Mean corpuscular hemoglobin
MCHC	Mean corpuscular hemoglobin concentration
PLT	Platelet
WBC	White blood cell
MO	Monocyte
LY	Lymphocyte
AST	Aspartate aminotransferase
ALT	Alanine aminotransferase
ALP	Akaline phosphatase
BUN	Bood urea nitrogen
CRE	Creatinine
PWT	Paw withdrawal threshold
PWL	Paw withdrawal latency
