# Supplementary material for: The Preparation and Evaluation of Carvacrol-Added Hyaluronic Acid for Early Osteoarthritis Treatment
Source: Antioxidants (Basel). 2025 Oct 21;14(10):1265. doi: 10.3390/antiox14101265 (PMC12561889; doi:10.3390/antiox14101265)
Supplement: Supplementary file 1 [file antioxidants-14-01265-s001.zip › antioxidants-3860756-supplementary.pdf]

Article

The Preparation and Evaluation of Carvacrol-added Hyaluronic Acid for Early Osteoarthritis Treatment

Yu-Ping Chen [1,2], Jhih-Ni Lin [2], Chia-Tien Chang [1,2], Yu-Ying Lin [1], Che-Yung Kuan [1], Yu-Chun Chen [3,*,†] and Feng-Huei Lin [1,2,4,*,†]

1. Doctoral Program in Tissue Engineering and Regenerative Medicine, National Chung Hsing University, Taichung 40227, Taiwan; asder89106@gmail.com (Y.-P.C.); aaiaaiaai0813@gmail.com (C.-T.C.); butter97132195@gmail.com (Y.-Y.L.); madkuan@gmail.com (C.-Y.K.)
2. Institute of Biomedical Engineering and Nanomedicine, National Health Research Institutes, Miaoli 35053, Taiwan; febe200630@gmail.com
3. Department of Chemical Engineering, College of Engineering and Science, National United University, Miaoli 36063, Taiwan
4. Department of Biomedical Engineering, College of Medicine and College of Engineering, National Taiwan University, Taipei 10617, Taiwan

* Correspondence: joycechen@nuu.edu.tw (Y.-C.C.); double@ntu.edu.tw (F.-H.L.); Tel.:+886-37-382185(Y.-C.C.); +886-37-206166 (ext. 37101) (F.-H.L.)

† These authors contributed equally to this work

Supplementary Materials

Table S1. Primers for RT-PCR performance.

Gene	Forward primer (5'-3')	Reverse primer (5'-3')
IL-1β	TGC TCT GGG ATT CTC TTC AG	TGT AGT GGT GGT CGA GAT T
IL-6	AGC CCA GCT ATG AAC TCC TT	CCT CAA ACT CCA AAA GAC CA
TNF-α	TCC TAC CAG ACC AAG GTC AA	TCG GCA AAG TCG AGA TAG TC
IL-1RA	ACA GCT GCC TGC AGT ACT TT	CGA GTC ATG TTT CCT GCT CT
iNOS	CCA GAA GCA GAA TGT GAC CA	GGA CCA GCC AAA TCC AGT C
CAT	TCT CAC CAA GGT TTG GCC TC	GCG GTG AGT GTC AGG ATA GG
SOD	ACA AAG ATG GTG TGG CCG AT	AAC GAC TTC CAG CGT TTC CT
GPX	GAA CCG TTC GCG GAG GAA AG	AGA GCG TGA ATG GGG CAT AG
Collagen I	CAG CCG CTT CAC CTA CAG C	TTT TGT ATT CAA TCA CTG TCT TGC C
Collagen II	GGC AAT AGC AGG TTC ACG TAC A	CGA TAA CAG TCT TGC CCC ACT T
Aggrecan	TCG AGG ACA GCG AGG CC	TCG AGG GTG TAG CGT GTA GAG A
Versican	TGG AAT GAT GTT CCC TGC AA	AAG GTC TTG GCA TTT TCT ACA ACA G
MMP 3	TCC CTC AGG AAG CTT GAA CCT GAA	AAA CCT AGG GTG TGG ATG CCT CTT
MMP 13	TGC TTC CTG ATG ACG ATG TAC	TCC TCG GAG ACT GGT AAT GG
GAPDH	GGA AGG ACT CAT GAC CAC AG	TTG GCA GGT TTT TCT AGA CG

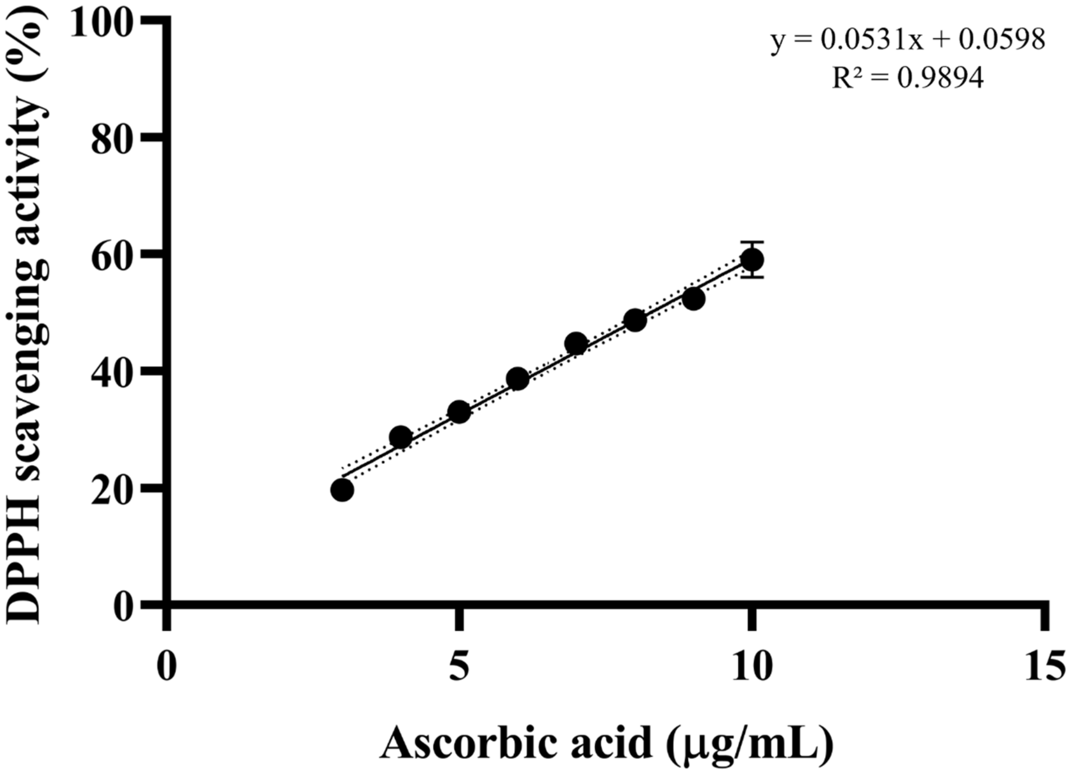

Figure S1. Ascorbic acid was used as a positive control in the DPPH assay. The scavenging activity increased in a dose-dependent manner across the tested concentration range, confirming the validity of the assay. This standard curve was used as a reference to interpret the antioxidant capacity of the HA–carvacrol formulation. Data are presented as mean ± SD (*n* = 6).

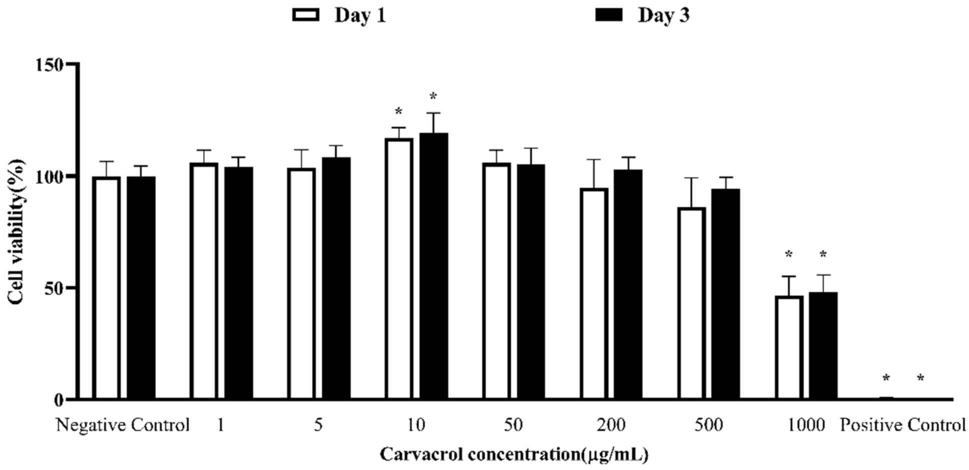

Figure S2. Cell viability of carvacrol at different concentrations in C20A4 cells evaluated by WST assay. The results demonstrated that 10 μg/mL provided the best cell viability and was therefore selected as the working concentration for subsequent experiments. Data are presented as mean ± SD ($n = 6$). * $p < 0.05$ compared with the negative control group on the same day.

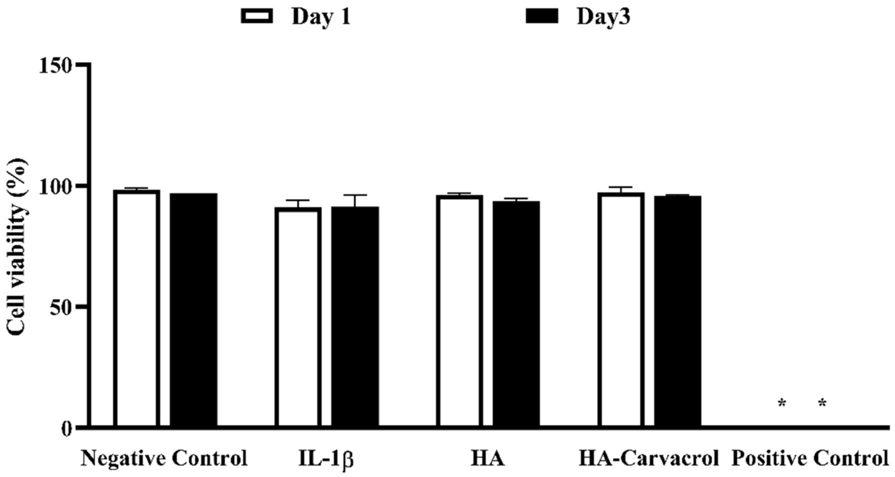

Figure S3. Quantitative analysis of live/dead staining in IL-1β-stimulated C20A4 chondrocytes. Live/dead assay indicated that HA-Carvacrol treatment maintained good cell viability under IL-1β stimulation. Data are presented as mean ± SD ($n = 6$). * $p < 0.05$ compared with the negative control group on the same day.
